# Influence of blood meal and age of mosquitoes on susceptibility to pyrethroids in *Anopheles gambiae* from Western Kenya

**DOI:** 10.1186/s12936-019-2746-6

**Published:** 2019-04-02

**Authors:** Maxwell G. Machani, Eric Ochomo, David Sang, Mariangela Bonizzoni, Guofa Zhou, Andrew K. Githeko, Guiyun Yan, Yaw A. Afrane

**Affiliations:** 1 Climate and Human Health Research Unit, Centre for Global Health Research, Kenya Medical Research Institute, Kisumu, Kenya; 2 School of Public Health and Community Development, Maseno University, Maseno, Kenya; 3 Entomology Section, Centre for Global Health Research, Kenya Medical Research Institute, Kisumu, Kenya; 4 Department of Biology and Biotechnology, University of Pavia, 27100 Pavia, Italy; 5 Program in Public Health, College of Health Sciences, University of California, Irvine, CA 92697 USA; 6 Department of Medical Microbiology, College of Health Sciences, University of Ghana, Accra, Ghana

**Keywords:** *Anopheles gambiae*, Feeding status, Age, Pyrethroid resistance, Kenya

## Abstract

**Background:**

Physiological characteristics (age and blood feeding status) of malaria vectors can influence their susceptibility to the current vector control tools that target their feeding and resting behaviour. To ensure the sustainability of the current and future vector control tools an understanding of how physiological characteristics may contribute to insecticide tolerance in the field is fundamental for shaping resistance management strategies and vector control tools. The aim of this study was to determine whether blood meal and mosquito age affect pyrethroid tolerance in field-collected *Anopheles gambiae* from western Kenya.

**Methods:**

Wild mosquito larvae were reared to adulthood alongside the pyrethroid-susceptible Kisumu strain. Adult females from the two populations were monitored for deltamethrin resistance when they were young at 2–5 days old and older 14–16 days old and whether fed or unfed for each age group. Metabolic assays were also performed to determine the level of detoxification enzymes. Mosquito specimens were further identified to species level using the polymerase chain reaction (PCR) method.

**Results:**

*Anopheles gambiae* sensu stricto was the predominant species comprising 96% of specimens and 2.75% *Anopheles arabiensis*. Bioassay results showed reduced pyrethroid induced mortality with younger mosquitoes compared to older ones (mortality rates 83% vs. 98%), independently of their feeding status. Reduced mortality was recorded with younger females of which were fed compared to their unfed counterparts of the same age with a mortality rate of 35.5% vs. 83%. Older blood-fed females showed reduced susceptibility after exposure when compared to unfed females of the same age (mortality rates 86% vs. 98%). For the Kisumu susceptible population, mortality was straight 100% regardless of age and blood feeding status. Blood feeding status and mosquito age had an effect on enzyme levels in both populations, with blood fed individuals showing higher enzyme elevations compared to their unfed counterparts (P < 0.0001). The interaction between mosquito age and blood fed status had significant effect on mosquito mortality.

**Conclusion:**

The results showed that mosquito age and blood feeding status confers increased tolerance to insecticides as blood feeding may be playing an important role in the toxicity of deltamethrin, allowing mosquitoes to rest on insecticide-treated materials despite treatment. These may have implications for the sustained efficacy of indoor residual spraying and insecticide-treated nets based control programmes that target indoor resting female mosquitoes of various gonotrophic status.

**Electronic supplementary material:**

The online version of this article (10.1186/s12936-019-2746-6) contains supplementary material, which is available to authorized users.

## Background

*Anopheles gambiae* sensu lato (s.l.) is the main vector of malaria in sub-Saharan Africa, where the disease is still a major public health problem causing significant morbidity and mortality despite concerted effort to control it [1]. Vector control and surveillance are the most important components of malaria control that aims at reduction and eventual interruption of the malaria parasite transmission cycle [2]. The core strategies employed for malaria vector control are the application of long-lasting insecticidal nets (LLINs) and the use of indoor residual spraying (IRS) [1].

A major challenge facing the use of insecticides for malaria vector control is the development of insecticide resistance. This has now been reported in almost all countries of sub-Saharan Africa and may continue to threaten the sustainability of malaria strategies [3, 4]. In Kenya, the main malaria vector, *An. gambiae* sensu stricto (s.s.) is already showing high levels of resistance to pyrethroid insecticides, which are the mainstay of vector control in the country [5, 6]. There is evidence that these levels of resistance may reduce the efficacy of treated bed nets and indoor residual spraying with pyrethroids [7, 8]. Two main resistance mechanisms have been identified in *An. gambiae* s.s. mosquitoes in Kenya: insecticide target site insensitivity achieved by point mutations that render the actual targets of an insecticide less sensitive to the active ingredient [9–11], and metabolic resistance involving the sequestration, metabolism, and/or detoxification of the insecticide, largely through the overproduction of specific enzymes [5, 12].

The current spread of pyrethroid resistance in major malaria vector *An. gambiae* s.s. in western Kenya emphasizes the need to investigate and understand its controlling factors and also the changing trends of insecticide resistance in target vectors and their consequence on malaria control. Under the field situation, vector control tools deployed target mosquito vectors of different age and nutritional differences (blood-fed and non-blood-fed) which may influence their effectiveness as malaria vector control tools. Some studies have shown that providing mosquitoes with a blood meal may decrease their sensitivity to insecticides [13–16]. For instance, some studies on *Anopheles stephensi*, *Aedes aegypti* and *Culex pipiens* have reported blood meal as one of the factors that may influence insecticide tolerance levels to dichlorodiphenyltrichloroethane (DDT) and dieldrin [17, 18]. Also, some studies have shown reduced phenotypic resistance with mosquito age [15, 16, 19–23]. Most of these earlier studies [13–15], linking physiological routes to insecticide resistance, have been performed using laboratory-reared mosquitoes, with few studies investigating how these link to natural populations. This study investigated the influence of mosquito age and blood meal on susceptibility to deltamethrin of field population of *An. gambiae* as one of the factors that may promote resistance, thus leading to inefficiency or ineffectiveness of an insecticide.

## Methods

### Mosquito population and sampling

Mosquito larvae were collected from Bungoma county (00.54057°N, 034.56410°E, altitude 1545 m above the sea level) in Western Kenya highlands. Previous studies have shown that *An. gambiae* s.s. is the predominant species in this area [5, 10] and deltamethrin is the main insecticide used for vector control [8, 24].

Immature stages of *An. gambiae* s.l. were collected from their natural breeding sites, such as ponds and puddles using the standard 350-ml dippers and hand pipettes and kept in plastic bottles. To avoid the collection of siblings, larvae were sampled randomly from different breeding sites. The aquatic stages were pooled together then transported to the insectary of the Centre for Global Health Research, Kenya Medical Research Institute (KEMRI) in Kisumu, Western Kenya, and reared under standard conditions (25 ± 2 °C; 80% ± 4% Relative Humidity with a 12 h: 12 h light/dark cycle).They were placed in spring water in small trays and reared on a mixture of tetramin (fish food) and brewer’s yeast provided daily. Upon pupation, individuals were collected and transferred to cages and allowed to emerge as adults. Emerging adults were provided with a 10% sugar solution until ready to be used for bioassay tests. *Anopheles gambiae* s.s. Kisumu strain, a reference insectary susceptible mosquito colony, was reared simultaneously in a separated room and handled in the same manner through all manipulations.

### Adult resistance bioassays

Mosquitoes were divided into young (2–5 days) and older females (14–16 days). The mosquitoes with different ages were either blood fed or unfed. Mosquitoes were allowed to feed to repletion on cow blood obtained from the abattoir through a membrane feeder. Fed mosquitoes were maintained on a 10% sugar solution and allowed to digest their blood for 8 h. The unfed and fed mosquitoes that were either young or old were subjected to the WHO susceptibility test. For each set of bioassays, five batches of 25 females from each group (wild population and laboratory susceptible strain mosquitoes): Four batches (100 mosquitoes) were subjected to insecticides susceptibility test against 0.05% deltamethrin at temperatures of 25 ± 2 °C and 70–80% relative humidity following the standard World Health Organization (WHO) tube test protocol [25] and test batch [25] exposed to untreated filter paper which served as a control. Briefly, resistant mosquitoes was defined as mosquitoes that survived 24 h after the end of the bioassay, and susceptible mosquitoes as the mosquitoes that were knocked down during the 60 min exposure time or that died within the 24 h recovery period. The knockdown time (KDT) of females was reported every 10 min during the 60 min exposure period. Mortality was scored after the 24 h recovery period. Mosquitoes that were knocked down after 1-h exposure and those that were alive after the 1-h exposure and still surviving 24 h later were collected and stored individually in 95% alcohol for subsequent molecular analysis.

### Species identification

Surviving and susceptible individuals from the field population exposed to the WHO tube bioassay were identified to species level. DNA was extracted using ethanol precipitation from legs and wings of mosquitoes samples. The two sibling species (*An. gambiae* s.s. and *Anopheles arabiensis*) of the *An. gambiae* s.l. species complex were distinguished using conventional PCR [26].

### Metabolic enzyme activity assays

Microplate enzyme system was used to quantify the levels of detoxifying enzymes (Monooxygenases, glutathione-*S*-transferase, and β-esterases) on individual female mosquitoes without insecticide exposure as previously described [27]. To ensure host components of blood meal could not interfere with the biochemical assays, 2 days and 13 days old female mosquitoes from the two populations were blood fed on cow using membrane feeder and allowed for 72 h to completely digest their blood meals. During the 72 h period, mosquitoes were maintained on a 10% sucrose. Therefore, in this experiment mosquitoes assayed were 5 days and 16 days old. After 72 h the mosquitoes were killed and frozen in individual tubes at − 80 °C. Mean absorbance values for each tested mosquito and enzyme were standardized based on the total protein amount. Total protein was measured for each mosquito as previously described [27]. All measurements were done in triplicates.

### Data analysis

The results of the 24-h WHO bioassays were analysed based on WHO criteria [25] and adjusted where necessary using abbots formula [28]. Knockdown rates were compared using Chi square. The effect of age and blood feeding on mortality rate, knockdown rate and enzyme activities were analysed using generalized linear model (GLM) with the inclusion of study site (strain) as covariate and interactions between age and blood fed status. In order to determine significant differences in enzymatic expression in each population (Field and Kisumu laboratory susceptible strain), mean absorbance values were compared between the unfed and blood fed unexposed individuals of different age groups (young and old) using analysis of variance (ANOVA) with a Tukey comparison-of-means as a post hoc test. All confidence intervals were set at 95%. Statistical analysis was performed using SPSS Version 21.

### Ethics statement

This study was approved by the Ethical Review Board of the Kenya Medical Research Institute (KEMRI) under the scientific steering committee (SSC 2267). For mosquito collection, oral consent was obtained from land owners in each location. These locations were not protected land, and the field studies did not involve endangered or protected species.

## Results

### Adult resistance bioassay

**Fig. 1:**
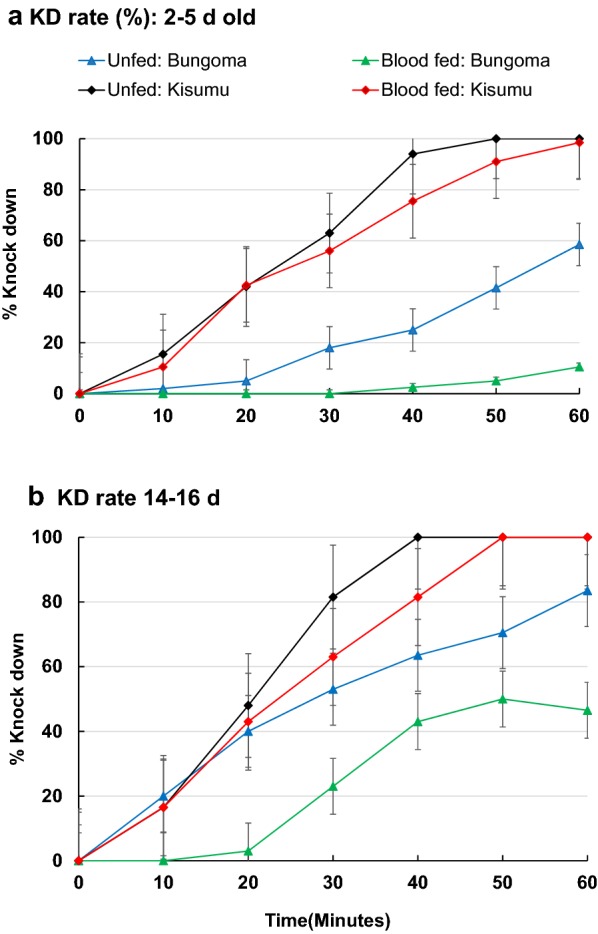
Percentage knockdown of young and old adult *Anopheles gambiae* during 60-min exposure to 0.05% deltamethrin. Panel **a** represents knockdown rates for young (2–5 days old) female mosquitoes and **b** knockdown rates for old (14–16 days old) female mosquitoes. Error bars indicate 95% confidence intervals

The field population showed delayed knockdown within the 60 min exposure to 0.05% deltamethrin compared to Kisumu strain (Table 1). Blood-fed female mosquitoes independent of age group recorded less than 50% knockdown within the 50 min exposure unlike their unfed counterparts from the same population (Fig. 1). Though Kisumu strain was knockdown within the 60 min exposure, induction of tolerance due to blood feeding was observed in blood-fed females that recorded less than 80% knockdown within the 40 min exposure, unlike their unfed counterparts that recorded 100% knockdown within the 40 min exposure independent of age group (Fig. 1).

**Table 1:** Comparison of knockdown curves of *Anopheles gambiae* with different physiological status (Age and feeding status) and population exposed to 0.05% deltamethrin (d* = days)

Population	Fixed factor		Comparing parameters	Chi square	d.f.	*P* value
Bungoma	Mosquito age					
	2–5 days		Blood-fed vs. unfed	9.08	5	0.0389
	14–16 days		Blood-fed vs. unfed	60.17	5	< 0.0001
	Feeding status					
	Unfed		2–5 days vs. 14–16 days	46.70	5	< 0.0001
	Blood fed		2–5 days vs. 14–16 days	17.19	4	0.0008
Kisumu	Mosquito age					
	2–5 days		Blood-fed vs. unfed	2.47	5	0.1502
	14–16 days		Blood-fed vs. unfed	4.76	5	0.1279
	Feeding status					
	Unfed		2–5 days vs. 14–16 days	9.12	5	0.0383
	Blood fed		2–5 days vs. 14–16 days	2.52	5	0.1508
Bungoma vs. Kisumu	Mosquito age					
	2–5 days		Fed vs. fed	23.99	5	< 0.0001
			Unfed vs. unfed	40.15	5	< 0.0001
	14–16 days		Fed vs. fed	43.94	5	< 0.0001
			Unfed vs. unfed	9.53	5	0.0334
	Feeding status					
	Blood fed		2–5 days vs. 2–5 days	23.99	5	< 0.0001
			14–16 days vs. 14–16 days	43.94	5	< 0.0001
	Unfed		2–5 days vs. 2–5 days	40.15	5	< 0.0001
			14–16 d vs. 14–16 days	9.53	5	0.0334

**Fig. 2:**
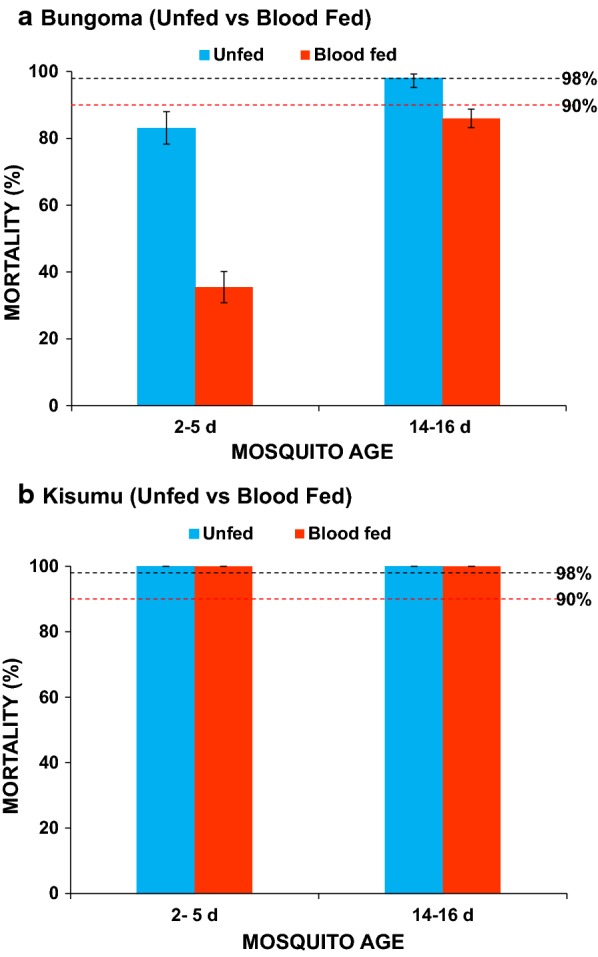
Mortality rates of wild *Anopheles gambiae* mosquitoes with different age groups and feeding status following exposure to deltamethrin (0.05%). Percentage mortalities for 24 h post deltamethrin exposure are given for **a** Bungoma population and **b** Kisumu strain. Dotted lines represent upper (98%) and lower (90%) cut-offs for WHO classifications; values above the upper line indicate susceptibility and values below the lower red line indicate resistance [25]. Error bars indicate 95% confidence intervals

Generalized linear model analysis found that mosquito age, blood fed status, and strains has significant effect on knock down rate when against deltamethrin (R^2^ = 0.92, adj R^2^ = 0.91, F_7,72_ = 113.30, P < 0.0001, Table 2). However, the interaction between age and blood fed status had no significant effect on knockdown rates (Table 2). There was significantly lower mortality in younger unfed mosquitoes aged 2–5 days compared to older unfed females aged 14–16 days (83% vs 98%; P < 0.0001; Fig. 2a). For fed mosquitoes, mortality rates for younger females (2–5 days) were far lower than older females aged 14–16 days (35.5% vs 86%; P < 0.0001; Fig. 2a).

**Table 2:** Coefficients (estimate) and significance of GLM analysis with Knockdown rates as dependent variable

Term	Estimate	t value	Pr (> |t|)
(Intercept)	46.50	14.88	< 0.0001
Site [Kisumu vs. Bungoma]	53.50	12.11	< 0.0001
Age [2–5 days vs. 14–16 days]	− 36.00	− 8.15	< 0.0001
Fed status [Unfed vs. Fed]	37.00	8.37	< 0.0001
Age * Fed status	11.00	1.76	0.0827
Site * Age	34.50	5.52	< 0.0001
Site * Fed status	− 37.00	− 5.92	< 0.0001
Site * Age * Fed status	− 9.50	− 1.08	0.2861

Interactions between variates were indicated by ‘*’

Younger blood-fed mosquitoes had much lower mortality rate as compared to the unfed mosquitoes of the same age (35.5% vs. 83%; P < 0.0001) whilst older fed mosquitoes also had much lower mortality rate as compared to the unfed mosquitoes of the same age (86% vs. 98%; P = 0.0225). The Kisumu strain which was used as a control, showed 100% susceptibility to deltamethrin independent of their age and feeding status.

Generalized linear model analysis revealed significant interaction effect between age and blood fed status on mortality (R^2^ = 0.92, adj R^2^ = 0.91, F_7,80_ = 130.56, P < 0.0001, Table 3). Clearly, when against deltamethrin, overall mortality of Bungoma strain mosquitoes was significantly lower than that of *An. gambiae* s.s. Kisumu strain, mortality of young mosquitoes was significantly lower than that of old mosquitoes, and blood feeding significantly reduced mortality (Table 3). There was a significant interaction effect between mosquito age and blood fed status on mosquito mortality (Table 3). There was also significant interaction effects on mortality between site and age, site and blood fed status, and among them (Table 3).

**Table 3:** Coefficients (estimate) and significance of GLM analysis with mortality as dependent variable

Term	Estimate	t Ratio	Prob > |t|
Intercept	87.81	127.63	<0.0001
Site [Bungoma vs. Kisumu]	− 12.19	− 17.71	<0.0001
Age [2–5 days vs. 14–16 days]	− 8.19	− 11.90	<0.0001
Fed status [fed vs. unfed]	− 7.44	− 10.81	<0.0001
Age [2–5 days] * Fed status [fed]	− 4.44	− 6.45	<0.0001
Site [Bungoma] * Age [2–5 days]	− 8.19	− 11.90	<0.0001
Site [Bungoma] * Fed status [fed]	− 7.44	− 10.81	<0.0001
Site [Bungoma] * Age [2–5 days] * Fed status [fed]	− 4.44	− 6.45	<0.0001

Interactions between variates were indicated by ‘*’

### Species composition

A sample of 400 *An. gambiae* s.l. exposed to WHO tube bioassays from field collections were processed by PCR to discriminate the sibling species. PCR analysis showed that *An. gambiae* s.s. was the predominant species in Bungoma population, comprising 384 (96.0%) with *An. arabiensis* being 11 (2.75%) and 5 (1.25%) did not amplify. Survived mosquitoes exposed to insecticide were all *An. gambiae* s.s.

### Metabolic enzymes activities

The monooxygenases, GSTs and β-esterases enzyme activity analyzed, were significantly elevated in the field population as compared to laboratory strain (F_1,313_ = 49.122, P < 0.0001, F_1,313_ = 37.942, P < 0.0001 and F_1,313_ = 82.182, P < 0.0001, respectively).

**Fig. 3:**
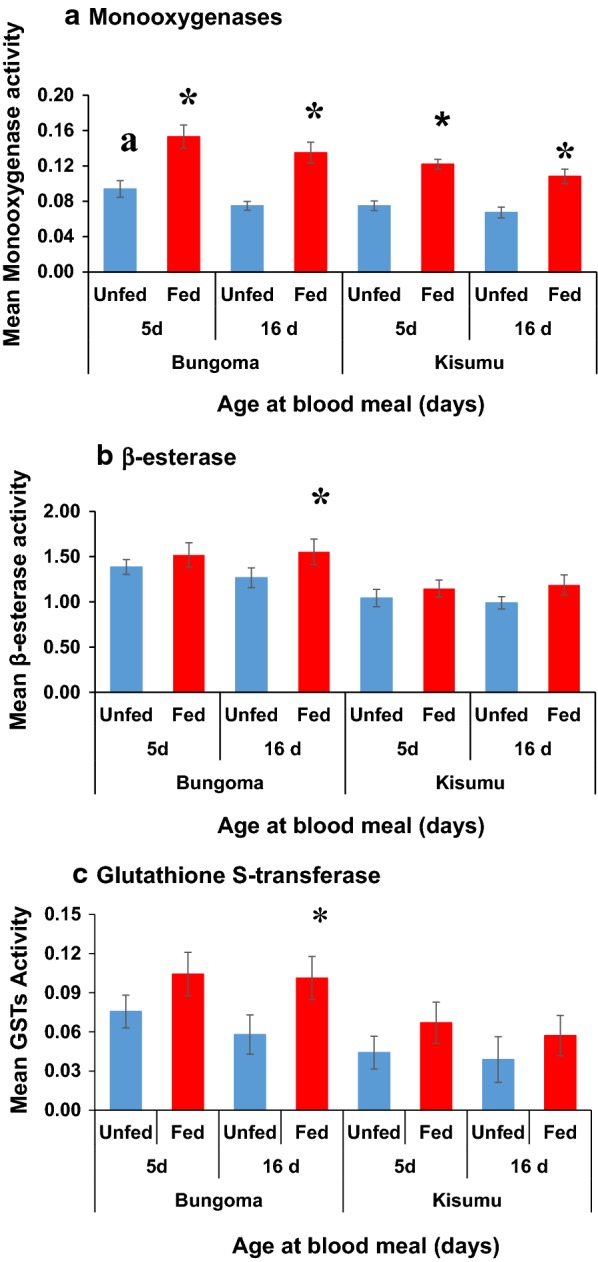
Enzyme activity levels (monooxygenases, β-esterases, and GSTs) measured in biochemical assays in both fields (Bungoma) population and Kisumu susceptible strain with different age groups and feeding status. Panel **a** represents monooxygenase activity, **b** esterase activity and **c** Glutathione S-transferase. Bars labelled with asterisks* indicates findings that are significantly different from others (Blood-fed vs. unfed individuals). The lower case letter (*a*) denotes a significant difference between younger unfed mosquitoes (5 days old) and Older (16 days old) unfed ones from the same populations. Error bars indicate 95% confidence intervals

Mosquito age had an effect on monooxygenases enzyme activity independent of mosquitoes abdominal status. The enzyme activity was significantly elevated in young (5 days old) field population compared to their older (16 days old) counterparts (F_1,313_ = 23.510, P < 0.0319, Fig. 3a). There was a slight elevation in β-esterases and GSTs enzyme activity in younger mosquitoes from the field population compared to older ones, though the enzyme elevation was not statistically significant (P > 0.05, Fig. 3b, c).

Figure 3 shows the changes in detoxification enzyme levels following a blood meal in females *An. gambiae* s.s. Monooxygenases activity was higher by 1.6- to 1.8-folds in blood-fed groups compared with unfed individuals from the same age groups and populations (Fig. 3a). Older blood-fed mosquitoes from the field population showed a significant increase of β-esterase and GSTs enzyme activities by 1.3- and 1.8-folds compared to unfed individuals of the same age and population (F_1,313_ = 23.344, P < 0.0032, and F_1,313_ = 27.897, P < 0.0014, respectively, Fig. 3b, c). These results indicate that both age and blood feeding were more likely to influence the different levels of the three enzyme activities tested.

For Kisumu susceptible strain, mosquito age and blood feeding did not have any significant change in the three enzymes analyzed, though there was increased enzyme activity by 1.2- to 1.6-fold change in blood fed groups compared to unfed individuals (P > 0.05, Fig. 3). GLM analysis indicated that site, mosquito age and blood fed status all had significant impact on mortality (Table 3). In addition, there were significant interaction effects on enzyme activities between mosquito age and blood fed status, between site and age, but not between site and blood fed status (Additional file 1).

## Discussion

Insects may survive the toxic effect of insecticides by different physiological mechanisms including target-site insensitivity and elevated detoxifying enzyme production [29, 30]. In western Kenya, pyrethroid resistance in *An. gambiae* s.s. is likely mediated by a combination of *kdr* and metabolic mechanisms as the primary mode of resistance [5, 8, 24, 31]. This study investigated the influence of blood meal status and mosquito age on susceptibility to deltamethrin in *An. gambiae* as some of the factors that can lead to ineffectiveness of insecticides used in malaria vector control.

In this study delayed knockdown for younger female mosquitoes exposed to 0.05% deltamethrin was recorded compared to their older counterparts independent of their feeding status, suggesting that younger mosquitoes may be able to forage on bed nets looking for hosts and rest on insecticide-treated walls for a longer period. This continuous exposure may affect both the overall mosquito responses to current insecticides used and the selection of resistance mechanisms which may compromise the effectiveness of the current malaria control efforts. A similar scenario has been observed in other studies on *An. stephensi* and *An. gambiae* showing a reduction in mean knockdown times of 10-day old adults, relative to newly emerged mosquitoes [19].

Field mosquitoes that were older and unfed (14–16 days old) which form that proportion of the population actively transmitting malaria, showed reduced phenotypic resistance compared to the younger 2–5 days old mosquitoes when exposed to deltamethrin. The mortality rates in the older unfed females were not different from the Kisumu laboratory strain that is considered susceptible to the insecticides used. This result shows that as mosquitoes age they become more sensitive to insecticides. The results were similar to previous studies on laboratory-colonized *An. gambiae* that have shown that sensitivity to insecticides increases as mosquitoes age [15, 16, 32].

The increase in insecticide susceptibility in these old unfed field-collected mosquitoes that showed increased phenotypic resistance in WHO susceptibility tests at younger age, might be a trade-off between energy used for defense against insecticides, onset of senescence, increase in the rate of cuticle permeability as some of the physiological changes that reduce the adaptation of mosquito to environmental changes [19, 32]. In this study, there was significant fluctuations of monooxygenase enzyme activity with mosquito age, which could also explain the increased mortality in the older unfed females. The fluctuations in enzyme activity in older mosquitoes could be due to the reduction of soluble proteins as the mosquito ages [19]. Previous studies carried out from the same Bungoma field sites have also linked pyrethroid resistance in the region with increased levels of detoxification enzymes [5].

Extensive use of insecticide for agricultural activity and public health purposes has been recognized to select for resistance phenotypes that may survive the available interventions [10, 33]. Vector longevity is an essential parameter in disease transmission as it increases the potential for infective bites to hosts [16, 34]. The reduced susceptibility observed in older fed females due to blood feeding indicates that this group can undergo insecticide pressure and survive long enough and continues with the transmission. This may also lead to behavioral modification as some of the older mosquitoes may shift their resting behaviour from indoor locations to outdoor locations away from the indoor interventions designed to target older females resting indoors.

Mortality rates were lower in the blood-fed mosquitoes in comparison to the unfed mosquitoes independent of age group. This indicates that the presence of blood meal reduced the effect of the insecticide on the mosquitoes even as the mosquitoes aged. The change in susceptibility in the blood-fed mosquitoes may possibly be due to the increased systemic expression of detoxification enzymes as metabolic activity increases during the process of blood digestion, which may also confer increased tolerance of mosquitoes to insecticides. A similar phenomena was observed by Spillings et al. [13], where tolerance of laboratory-reared female *Anopheles funestus* to pyrethroids increased about two-fold at 24 h after a blood meal. Oliver and Brooke [14] also observed significantly reduced mortality in young and older blood fed laboratory reared *An. arabiensis* though in this study *An. arabiensis* identified after exposure were susceptible independent of their age and feeding status.

Mosquitoes feeding status and age evaluated in this study were shown to have an effect in enzyme systems. The three enzyme activities (monooxygenase, β-esterase, and GSTs) were elevated in older blood-fed female mosquitoes unlike the unfed older mosquitoes of the same population. The results demonstrate that blood feeding in field-collected female mosquito induced the overproduction of detoxification enzymes, which might have reduced deltamethrin-induced mortality, unlike their unfed counterparts. The increase in deltamethrin-induced mortality with age in the unfed group suggests that without increased enzyme activity induced by blood meal other mechanisms involved may become less efficient with age. Similar effects were observed in *An. arabiensis*, where blood meal induced a variable effect on mortality, with the younger and older blood-fed females showing significantly reduced mortality to deltamethrin [14].

Acquisition of blood meal for reproduction by female mosquitos has been shown to involve a complex series of biological events initiated in order to utilize the blood nutrients, with many transcriptional changes peaking after some few hours of feeding and which may last for 72 h with optimal conditions, this may present metabolic changes and induce oxidative stress [35]. Increased metabolic activity during the process of blood digestion has been shown to alter levels of different detoxification enzymes in mosquito midgut [36]. Such changes may have an impact on characteristics that affect vector control. For instance, The release of reactive oxygen species (ROS) during the digestion of blood meal (haem) can be toxic and has been shown to intensify after mosquito ingesting *Plasmodium* infected blood meal [37, 38]. This has been shown to increase systematic expression of several detoxifying enzymes some of which have been implicated in insecticide detoxification [30, 37]. Earlier studies on *Aedes aegypti* [39] and *Culex pipiens* [40] have shown expressions of genes altered after a blood meal, these genes have been linked to insecticide detoxification enzymes. Over-expression of CYP6M2 one of the cytochrome P450s genes has been reported in response to *Plasmodium* infected blood meal in both the midgut and the fat body of *An. gambiae* after 1 day of blood meal ingestion [41, 42]. Although this study did not measure the gene expressions levels, these could possibly explain the differential effects on the relative levels of insecticide resistance between the unfed and blood fed individuals from the same groups.

Given the criteria used to evaluate for resistant phenotypes in field populations, there is a need to consider blood-fed female mosquitoes as they may show enhanced expression of the resistance phenotypes. Also considering the scaling-up of long-lasting insecticidal nets incorporated with a synergist (enzyme inhibitor) for malaria vector control, for instance piperonyl butoxide (PBO) treated bed nets which has been shown to be more effective compared to conventional LLINs [43, 44] would be beneficial to control such blood fed individuals expressing a high level of metabolic enzymes.

## Conclusion

The results showed that age had an influence on mosquito susceptibility status to insecticides, with the younger individuals being resistant than the older individuals of the same population. Blood feeding also in vector mosquitoes plays an important role in the toxicity of deltamethrin. These may have implications for the sustained efficacy of the indoor residual spraying and insecticide-treated nets based control programmes that target indoor resting female mosquitoes of various gonotrophic status.

## Additional file

**Additional file 1: Table S1.** GLM analysis of enzymes.

## Abbreviations

LLINs: long-lasting insecticide treated nets
KDR: knockdown resistance
KDT: knockdown time
PCR: polymerase chain reaction
GLM: generalized linear model
ANOVA: analysis of variance
GSTs: glutathione-*S*-transferase
ROS: reactive oxygen species
SSC: scientific steering committee
PBO: piperonyl butoxide
