# Supplementary material for: Influence of blood meal and age of mosquitoes on susceptibility to pyrethroids in Anopheles gambiae from Western Kenya
Source: Malar J. 2019 Apr 2;18:112. doi: 10.1186/s12936-019-2746-6 (PMC6444593; doi:10.1186/s12936-019-2746-6)
Supplement: Supplementary file 1 — Additional file 1: Table S1. GLM analysis of enzymes. [file 12936_2019_2746_MOESM1_ESM.docx]

Table S1. GLM analysis of enzymes

| Enzyme | Oxidases | | Esterases | | GST | |
| --- | --- | --- | --- | --- | --- | --- |
| R^2^, adjusted | 0.32, adj 0.31 | | 0.49, adj 0.48 | | 0.38, adj 0.37 | |
| F_7,523_ | 35.67 |  | 71.55 |  | 45.11 |  |
| P-value | <0.0001 |  | <0.0001 |  | < 0.0001 |  |
| Regression terms | Estimate | Pr(>\|t\|) | Estimate | Pr(>\|t\|) | Estimate | Pr(>\|t\|) |
| (Intercept) | 0.1750 | < 0.0001 | 1.6932 | < 0.0001 | 0.1187 | < 0.0001 |
| Site [Kisumu] | -0.0584 | 0.0193 | -0.3862 | < 0.0001 | -0.0467 | < 0.0001 |
| Age [2d] | 0.2118 | < 0.0001 | 0.7416 | < 0.0001 | 0.0626 | < 0.0001 |
| Fed status [Unfed] | -0.0802 | 0.0037 | -0.3095 | 0.0003 | -0.0287 | 0.0052 |
| Age [2d] : Fed status [Unfed] | -0.1389 | 0.0002 | -0.2452 | 0.0308 | -0.0330 | 0.0165 |
| Site [Kisumu] : Age [2d] | -0.2046 | < 0.0001 | -0.7557 | < 0.0001 | -0.0548 | < 0.0001 |
| Site [Kisumu] : Fed status [Unfed] | 0.0347 | 0.3721 | 0.0770 | 0.5197 | 0.0025 | 0.8648 |
| Site [Kisumu] : Age [2d] : Fed status [Unfed] | 0.1415 | 0.0084 | 0.3422 | 0.0381 | 0.0360 | 0.0717 |
